# An unexpected abundance of bidirectional promoters within *Salmonella* Typhimurium plasmids

**DOI:** 10.1099/mic.0.001339

**Published:** 2023-05-19

**Authors:** Alistair D. Middlemiss, Emily A. Warman, David Forrest, James R. J. Haycocks, David C. Grainger

**Affiliations:** ^1^​ School of Biosciences, University of Birmingham, Edgbaston, Birmingham B15 2TT, UK

**Keywords:** cappable-seq, pervassive, RNA polymerase, RNA-seq, transcription

## Abstract

Transcription of the DNA template, to generate an RNA message, is the first step in gene expression. The process initiates at DNA sequences called promoters. Conventionally, promoters have been considered to drive transcription in a specific direction. However, in recent work, we showed that many prokaryotic promoters can drive divergent transcription. This is a consequence of key DNA sequences for transcription initiation being inherently symmetrical. Here, we used global transcription start site mapping to determine the prevalence of such bidirectional promoters in *Salmonella* Typhimurium. Surprisingly, bidirectional promoters occur three times more frequently in plasmid components of the genome compared to chromosomal DNA. Implications for the evolution of promoter sequences are discussed.

## Introduction

**Fig. 1.**
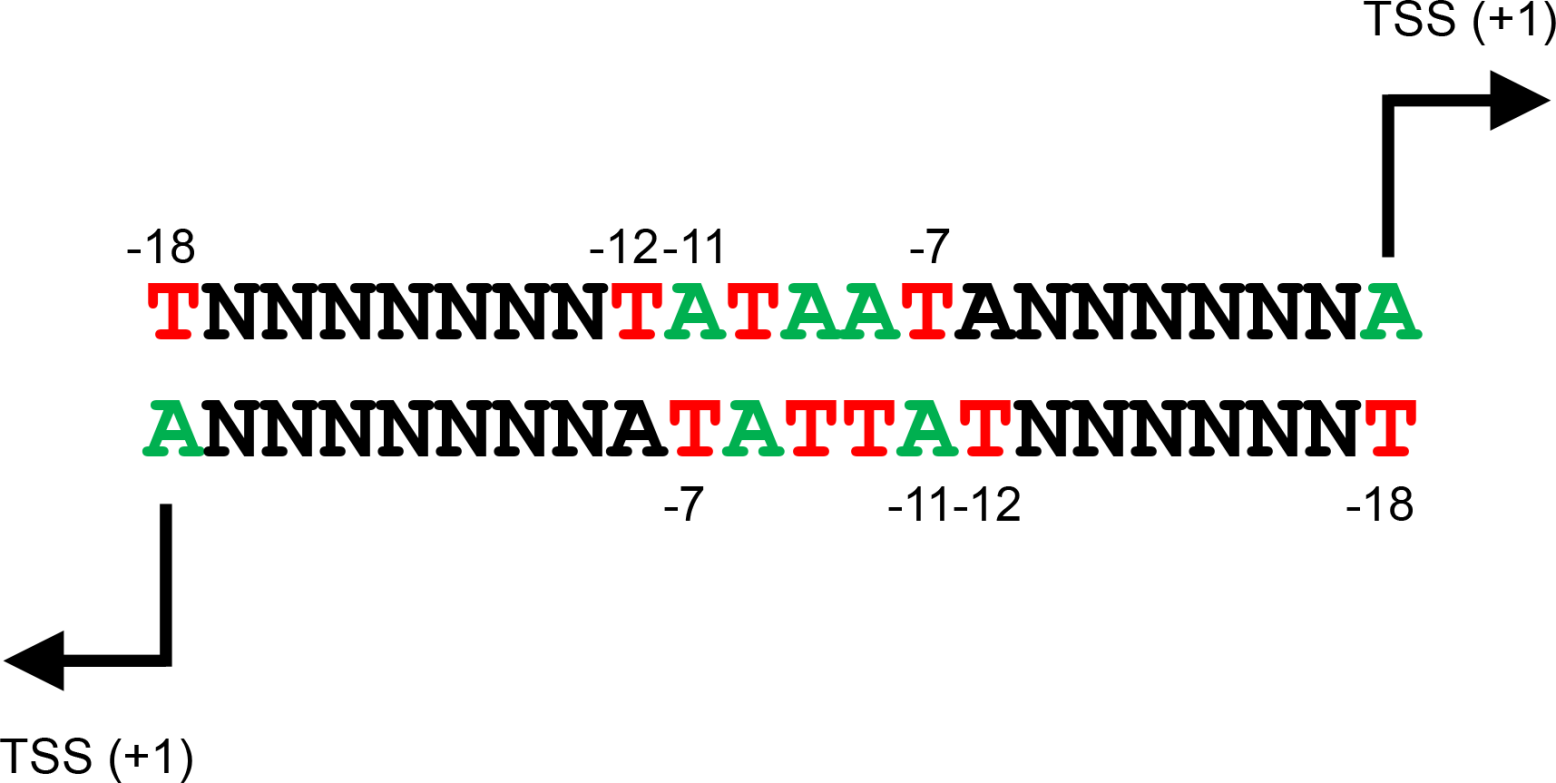
Organization of a bidirectional promoter sequence. The figure illustrates the most common bidirectional promoter configuration found in most bacteria [4]. Transcription start sites (TSSs) on opposite DNA strands are separated by 18 bp. Since the initiating nucleotide is most frequently ATP, a thymine base occurs most often at position −18 relative to each TSS. The −18T sequence alters DNA bending to stabilize interactions between the nucleic acid backbone and σ^70^ residue R451^10^. Even in the absence of other contacts, this is sufficient to stabilize DNA binding by RNA polymerase so that transcription can initiate. When TSSs are separated by 18 bp, the key promoter −10 element sequences, −7T and −11A, are reciprocally base paired on each DNA strand. These are key, and highly conserved in bacterial promoters, because both flip out of the base stack during DNA unwinding to interact with the RNA polymerase σ^70^ subunit [9]. Consequently, DNA regions such as those illustrated can bind RNA polymerase in either orientation to support transcription initiation.

The instructions encoded by DNA are organized into discrete packets of information called genes [1]. To utilize genes, the DNA code is copied to make an RNA message in a process called transcription. Promoters are segments of dsDNA to which RNA polymerase can specifically bind and initiate transcription [2]. For decades, promoters were considered to drive transcription in an explicit direction, usually to control the expression of a downstream gene [3]. However, it is now evident that promoters throughout the domains of life can often act bidirectionally [4–8]. This may result in an mRNA message being generated in one direction. However, the divergent RNA is often antisense to a gene and non-coding. In bacteria, promoter bidirectionality results from near symmetry of the promoter −10 element (5′-TATAAT-3′) [4]. Small deviations from the consensus permit sufficient symmetry for divergent transcription to initiate. In this scenario, two transcription start sites (TSSs) are usually found 18 bp apart on opposite DNA strands (Fig. 1). Whilst other spatial arrangements can occur, this organization is most frequent, in most bacterial species, for two reasons [4]. First, because of key interactions with RNA polymerase, positions −7 and −11, relative to the TSS, are the best conserved promoter sequences [9]. When divergent TSSs are separated by 18 bp, positions −7 and −11, relative to each TSS, reciprocally coincide (Fig. 1). Second, the initiating nucleotide (that equates to position +1) is usually ATP. Consequently, the base on the opposite DNA strand, at position −18 relative to the divergent TSS, is usually thymine (Fig. 1). This is beneficial because, even in the absence of transcriptional activators, and other promoter elements, a thymine at position −18 is sufficient to stimulate transcription [4, 10, 11].

In this work, we have mapped TSSs genome wide in *Salmonella* Typhimurium strain SL1344 using cappable-seq [12]. We identify 17 189 unique TSSs and these will serve as a useful resource for future studies of *S*. Typhimurium gene control. Consistent with our prior work, we detect thousands of transcripts resulting from divergent transcription at bidirectional promoters. Surprisingly, such transcripts are found to originate much more frequently from plasmid, rather than chromosomally, encoded promoters. Previously, we speculated that recently acquired DNA sequences are more likely to contain bidirectional promoters. This model is discussed with reference to our current observations.

## Methods

### Cappable-seq

Cappable-sequencing (cappable-seq) experiments were done as previously described [13, 14]. Briefly, *S*. Typhimurium strain SL1344 was grown to mid-log phase in LB medium and RNA was extracted using the SV Total RNA Isolation System (Promega). Generation of cappable-seq libraries, and Illumina sequencing, was done by Vertis Biotechnologie AG on 5 µg of extracted RNA. Raw sequencing reads are available from ArrayExpress using accession number E-MTAB-12506. The analysis was done twice and those TSSs detected were present in both replicates.

### Bioinformatics

Sequencing reads were mapped to the *S*. Typhimurium SL1344 reference genome (FQ312003.1, NC_017718.1, NC_017719.1 and NC_017720.1) using Bowtie2 and SAMtools (version 1.3.1). TSSs were identified using the software of Ettwiller *et al*. [12] Briefly, bam2firstbasegtf.pl was used to generate the .gtf files and relative read scores (RRSs). The latter represents the number of reads normalized to the total number of reads in the sample. The results are then filtered based on a cut-off value or 1.5 (equivalent to 20 reads or more). DNA sequence motifs were generated using weblogo [15]. The false positive rate of TSS detection by cappable-seq is 1.4 % [12].

## Results

### Genome-wide identification of TSSs in *S*. Typhimurium SL1344

The 5′ end of primary RNA transcripts is triphosphorylated. Conversely, processed RNAs have monophosphorylated 5′ ends. Hence, methods for identifying TSSs aim to identify triphosphorylated RNA termini. Many previous TSS mapping studies relied on the use of Xrn1 exonuclease that preferentially degrades RNAs with a monophosphorylated 5′ end. However, RNA folding can prevent nuclease access to its target. Consequently, RNA samples with and without Xrn1 treatment are compared. This is known as differential RNA-seq (dRNA-seq). More recently, cappable-seq has been used to identify TSSs. This method exploits the ability of vaccinia capping enzyme to specifically modify triphosphorylated RNA 5′ ends with biotinylated GTP. The reaction is highly specific, allows primary unprocessed transcripts to be isolated using streptavidin beads, and removes the need to generate a second sample for comparison. We used cappable-seq to map TSSs in *S*. Typhimurium SL1344. This strain has a 4 878 012 bp chromosome and two plasmids, pCol1B9 and pSLT, that are 86 908 and 93 842 bp in length respectively. Our isolate lacks plasmid pRSF1010 described in prior work [16]. We identified 17 189 TSSs genome-wide with 2417 of these being derived from pCol1B9 or pSLT. It is notable that the plasmids account for only 3.6 % of the genomic DNA but 14.1% of all detected TSSs. The list of TSSs detected by cappable-seq is provided in Table S1 (available in the online version of this article).

### Cappable-seq identifies the majority of TSSs identified by dRNA-seq

**Fig. 2.**
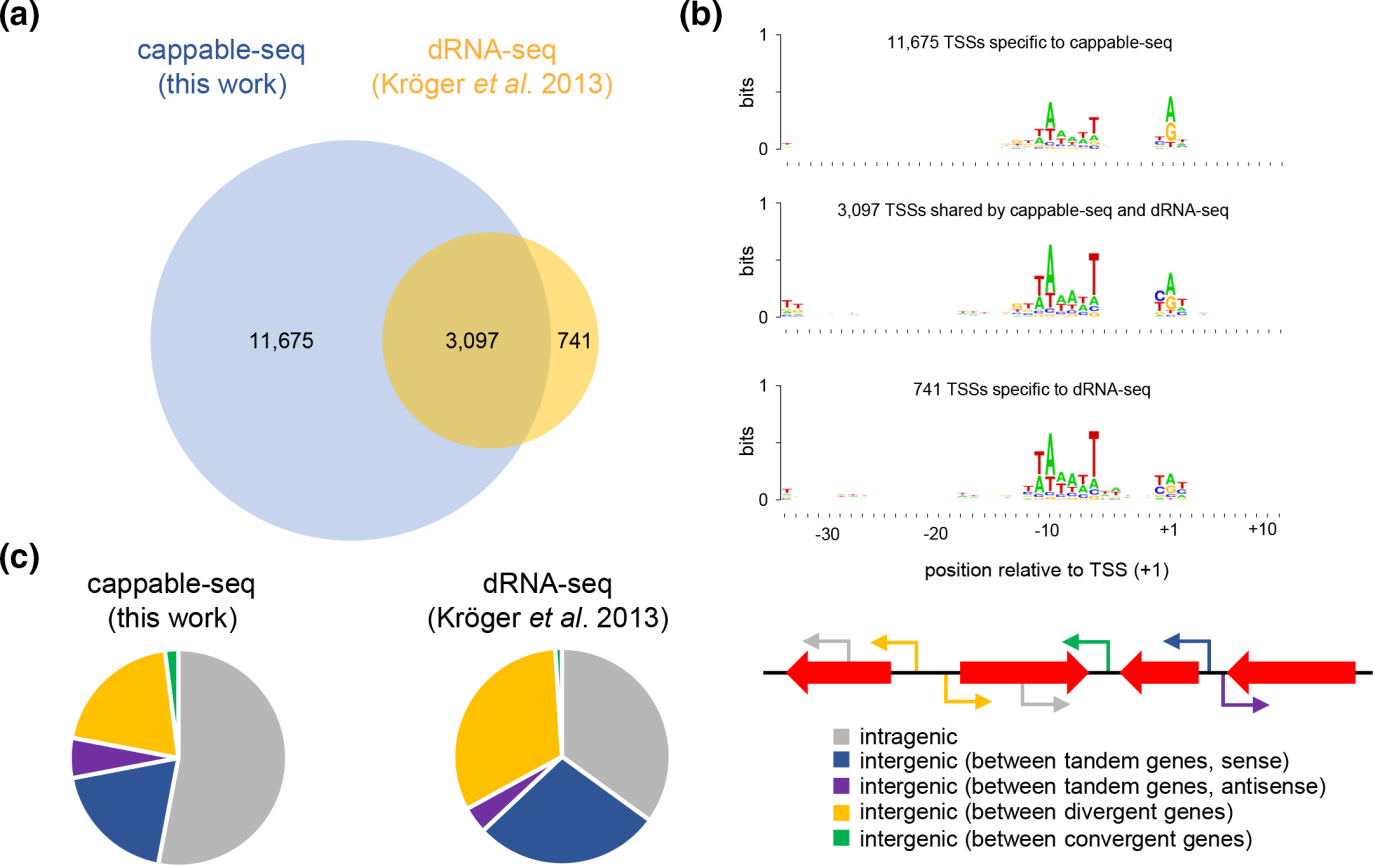
Identification and distribution of transcription start sites (TSSs) in *Salmonella* Typhimurium SL1344. (**a)** Cappable-seq identifies most TSSs identified by dRNA-seq. The Venn diagram illustrates overlap between TSSs identified by cappable-seq and dRNA-seq [17]. (**b)** Promoter sequences identified by different TSS mapping methods are similar. Promoter DNA sequences corresponding to each sector of the Venn diagram in (a) were aligned according to location of the TSS, and DNA sequence logos were generated. (**c)** Distribution of chromosomal TSSs according to genomic context. The pie charts indicate the location of all chromosomal TSSs (Kröger *et al*. did not report plasmid-derived RNAs) identified by cappable-seq and dRNA-seq. The different genomic contexts are illustrated by the schematic in which block arrows represent genes and line arrows depict promoters. Note that we did not differentiate between intragenic promoters in the sense or anti-sense orientation.

Previously, Kröger and co-workers used dRNA-seq to map chromosomal TSSs in the same strain and identified 3 838 RNA 5′ ends [17]. Of these TSSs, 3097 were also detected by our analysis. Thus, a total of 11 675 chromosomal TSSs were found only by cappable-seq and 741 only by dRNA-seq (Fig. 2a). For each of these three groups we aligned the promoter DNA sequences surrounding the TSSs. The alignments were then used to generate DNA sequence logos (Fig. 2b). As expected, all logos illustrated a preference for a purine at the TSS, and strong conservation of the promoter −10 element. Conversely, the −35 element is poorly conserved. This is a common feature of bacterial promoters and allows for regulation of initial RNA polymerase binding by transcriptional activators [18]. There are only small differences between the motifs. For example, TSSs specific to the cappable-seq experiment had stronger conservation of the purine at position +1 whilst TSSs detected by both capable-seq and dRNA-seq had marginally better conservation of the −35 hexamer. For further comparison, TSS locations with respect to gene sequences were determined for the cappable-seq and dRNA-seq datasets (Fig. 2c). For both analyses, TSSs were identified most frequently within coding DNA. However, the proportion of intragenic TSSs identified by cappable-seq (53 %) was higher than with dRNA-seq (34 %).

### Many promoters are bidirectional and drive divergent transcription

**Fig. 3.**
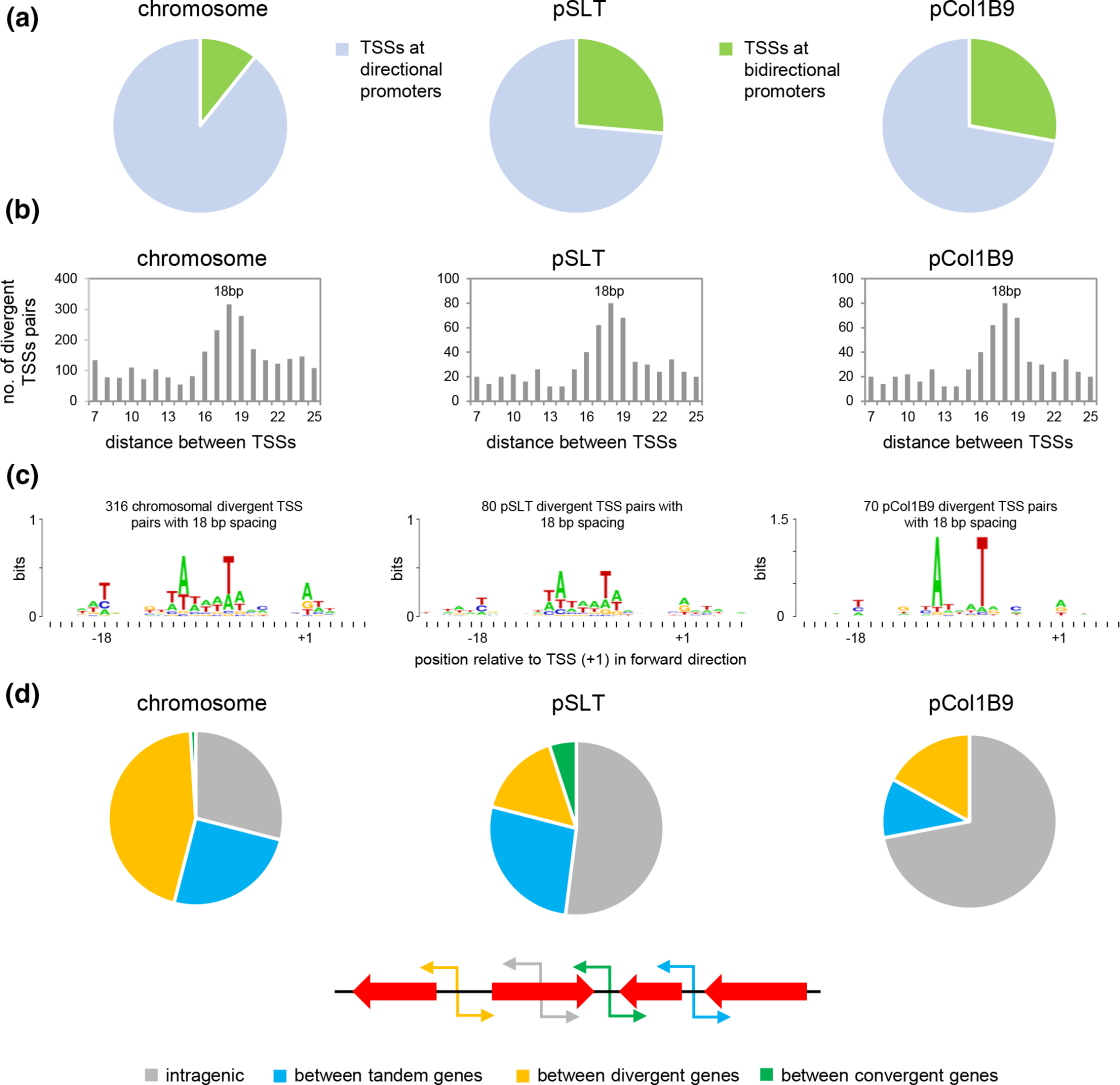
Bidirectional promoters are found most frequently within the plasmids of *Salmonella* Typhimurium SL1344. (**a)** Divergent transcription start site (TSS) pairs at bidirectional promoters are more frequently associated with plasmid DNA. TSSs associated with the chromosome, or plasmids pSLT and pCol1B9, were defined as originating from a bidirectional promoter if they were separated by between 7 and 25 bp on opposite DNA strands. (**b)** Spacing optima between divergent TSS pairs at bidirectional promoters. The histograms show the number of TSSs, separated by each distance between 7 and 25 bp, for each component of the genome. (**c)** DNA sequences associated with divergent TSS pairs separated by 18 bp. Sequences were aligned according to the position of the TSS in the forward direction. (**d)** Distribution of bidirectional promoters according to genomic context. The different genomic contexts are illustrated by the schematic in which block arrows represent genes and line arrows depict promoters.

Previously, we defined bidirectional promoters as being associated with divergent pairs of TSSs separated by between 7 and 25 bp [4]. In such instances, key positions in the promoter −10 element and TSS reciprocally overlap on opposite DNA strands [4]. Using these criteria, 2238 of the 17 189 TSSs identified were part of a divergent pair. Remarkably, of the 2238 divergent pairs of TSSs, 656 mapped to pCol1B9 and pSLT. Consequently, whilst 10.7 % of chromosomal TSSs formed part of a divergent TSS pair, the equivalent values for pSLT and pCol1B9, 26.4 and 27.8 % respectively, were far higher (Fig. 3a). As noted above, the number of all TSSs mapping to pSLT and pCol1B9 was disproportionately high. This bias is greatly exaggerated for divergent TSS pairs. As previously reported for *Escherichia coli *, most bidirectional promoters allowed transcription initiation at sites separated by 18 bp on opposite DNA strands (Fig. 3b).

### Bidirectional promoter sequence properties and genomic context

We selected DNA sequences corresponding to all sites of divergent transcription initiation separated by 18 bp. These sequences were aligned, according to the position of the TSS in the forward orientation, and used to generate DNA sequence logos (Fig. 3c). As expected, for both chromosomal and plasmid-derived sequences, position −18T on the top DNA strand was conserved, corresponding to the start site of transcription in the reverse direction (as illustrated in Fig. 1). Bidirectional promoters encoded by the chromosome and plasmid pSLT generated very similar DNA sequence logos. The motif for pCol1B9-encoded bidirectional promoters exhibited more prominent conservation of positions −7 and −11 relative to the TSS in the forward direction. Finally, we determined the position of bidirectional promoters with respect to genes. For chromosomal loci, bidirectional promoters were strongly biased towards intergenic regions, particularly those between divergent genes (Fig. 3d, left panel). Conversely, plasmid-borne bidirectional promoters were more likely to be found within coding DNA (Fig. 3d, middle and right panels).

## Discussion

We report high-resolution mapping of TSSs in *S*. Typhimurium SL1344 using cappable-seq. Our analysis identifies over 80 % of TSSs previously defined by Kröger *et al*. in the same organism [17] (Fig. 2a). We note that the compendium of TSSs reported previously were collected under 22 different growth conditions related to infection. Hence, we did not expect to recover all previously defined TSSs in a single growth condition not related to infection. It is notable that cappable-seq has greater sensitivity than dRNA-seq, identifying an additional 11 675 TSSs in this instance (Fig. 2a). This is consistent with comparison of similar datasets for other organisms [4]. The improvement is probably due to technical differences between the approaches. Most notably, in cappable-seq, primary RNA 5′ ends are directly tagged, permitting their specific enrichment and concomitant reduction in signal due to processed RNAs [12]. Whilst cappable-seq identified more TSSs than dRNA-seq in all genomic locations, this was most evident for transcripts initiating within genes and between convergent genes (Fig. 2c). This is probably because such transcripts are less stable, created less frequently and more easily confused with RNAs resulting from degradation of primary RNAs. As discussed above, this distinction is easier to make using cappable-seq. Although these mysterious RNA species remain poorly defined, roles have started to emerge [19]. In recent work, Figueroa-Bossi *et al*. demonstrated that non-coding intragenic transcription provides stochastic relief from gene silencing [20]. In other cases, defined functions go beyond the control of gene expression. For instance, in their work, Martinez and colleagues demonstrated that ‘pervasive’ transcription of the genome is important for transcription coupled DNA repair (TCR) [21]. Hence, parts of the chromosome not associated with mRNA production (intergenic regions and the DNA non-template strand) remain subject to TCR.

Importantly, promoter sequences associated with RNAs specifically detected by either cappable-seq or dRNA-seq are very similar (Fig. 2b). This is consistent with these RNA 5′ ends resulting from genuine transcription initiation, rather than RNA processing. Indeed, for cappable-seq, conservation of the TSS sequence was more pronounced for those RNAs not also detected by dRNA-seq (Fig. 2b). As noted above, this group of RNAs includes many transcripts located within genes and non-coding DNA.

The most surprising aspect of our analysis is the 3-fold enrichment for bidirectional promoters within plasmid components of the *S*. Typhimurium genome (Fig. 3a). This was particularly unexpected because the DNA sequence of these plasmids does not have an elevated AT content, which we have shown results in many promoter-like elements arising [22–24]. Previously, we and others suggested bidirectional promoters represent an early step in the evolution of sites for transcription initiation. Hence, they are found more frequently within horizontally acquired DNA sequences [4, 25]. In this regard, it is interesting to note differences in bidirectional promoter location when comparing chromosomal and plasmid loci (Fig. 2g). In the chromosome, bidirectional promoters are most frequently found between divergent genes where two mRNAs can be generated, as we also reported previously for *E. coli* [4]. Conversely, for pSLT and pCol1B9, bidirectional promoters are usually found within genes and, even when only intergenic regions are considered, there is no clear bias towards those between divergent genes (Fig. 2f). We speculate that evolution gradually removes or alters bidirectional promoters to avoid problematic transcription. Conversely, plasmids, by virtue of their ability to move into different host cells, are less susceptible to such changes.

## Supplementary Data

Supplementary material 1
